# Identification of microRNAs from Medicinal Plant *Murraya koenigii* by High-Throughput Sequencing and Their Functional Implications in Secondary Metabolite Biosynthesis

**DOI:** 10.3390/plants11010046

**Published:** 2021-12-24

**Authors:** Claudia Gutiérrez-García, Shiek S. S. J. Ahmed, Sathishkumar Ramalingam, Dhivya Selvaraj, Aashish Srivastava, Sujay Paul, Ashutosh Sharma

**Affiliations:** 1 Tecnologico de Monterrey, Centre of Bioengineering, School of Engineering and Sciences, Queretaro CP 76130, Mexico; A00887520@itesm.mx; 2 Omics and Drug Discovery Lab, Faculty of Allied Health Sciences, Chettinad Academy of Research and Education, Kelambakkam 603103, India; shiekssjahmed@gmail.com; 3 Plant Genetic Engineering Laboratory, Department of Biotechnology, Bharathiar University, Coimbatore 641046, India; rsathish@buc.edu.in (S.R.); bioinfodhivya@gmail.com (D.S.); 4 Section of Bioinformatics, Clinical Laboratory, Haukeland University Hospital, 5021 Bergen, Norway; aashish.srivastava1302@gmail.com; 5 Department of Clinical Science, University of Bergen, 5021 Bergen, Norway

**Keywords:** microRNA (miRNA), medicinal plant, *Murraya koenigii*, secondary metabolites, gene regulation, Illumina sequencing

## Abstract

MicroRNAs (miRNAs) are small noncoding RNA molecules that play crucial post-transcriptional regulatory roles in plants, including development and stress-response signaling. However, information about their involvement in secondary metabolism is still limited. *Murraya koenigii* is a popular medicinal plant, better known as curry leaves, that possesses pharmaceutically active secondary metabolites. The present study utilized high-throughput sequencing technology to investigate the miRNA profile of *M. koenigii* and their association with secondary metabolite biosynthesis. A total of 343,505 unique reads with lengths ranging from 16 to 40 nt were obtained from the sequencing data, among which 142 miRNAs were identified as conserved and 7 as novel miRNAs. Moreover, 6078 corresponding potential target genes of *M. koenigii* miRNAs were recognized in this study. Interestingly, several conserved and novel miRNAs of *M. koenigii* were found to target key enzymes of the terpenoid backbone and the flavonoid biosynthesis pathways. Furthermore, to validate the sequencing results, the relative expression of eight randomly selected miRNAs was determined by qPCR. To the best of our knowledge, this is the first report of the *M. koenigii* miRNA profile that may provide useful information for further elucidation of the involvement of miRNAs in secondary metabolism. These findings might be crucial in the future to generate artificial-miRNA-based, genetically engineered *M. koenigii* plants for the overproduction of medicinally highly valuable secondary metabolites.

## 1. Introduction

*Murraya koenigii* (L.) Spreng (*Rutaceae*) is a subtropical medicinal plant native to Asia and distributed throughout the subcontinent of India [1]. This plant has a great commercial value since it produces several important therapeutic compounds, including carbazole alkaloids [2] (only a few plant species can produce carbazole alkaloids, and the most significant one is *M. koenigii*). Different parts of the *M. koenigii* plant are widely used for treating a variety of ailments such as fever, diarrhea, diabetes, skin eruptions, venomous bites, renal pain, inflammation, and cholesterol management, among others, and have been used in Ayurvedic, Unani, and homeopathic medicine for centuries [3,4]. However, the leaf (curry leaf) of *M. koenigii*, which has long been used in Indian cuisine, is the most pharmacologically studied plant part due to its higher phytochemical contents. Consequently, numerous investigations have focused on studying and validating its pharmacological properties, such as antifungal, antibacterial, anti-inflammatory, antioxidant, anticancer, neuroprotective, and hepatoprotective [4]. For example, a recent study showed that hydroalcoholic extract of curry leaf suppressed several inflammatory and nitrosative stress markers, including nitrotyrosine (NT), cyclooxygenase-2 (COX-2), interleukin 1 beta (IL-1β), and intercellular adhesion molecule 1 (ICAM1), and increased the expression of nuclear factor erythroid 2-related factor 2 (Nrf-2), an important antioxidant protein in acute pancreatitis [5]. Additionally, the curry leaves’ methanolic and aqueous extracts were found to be nephroprotective against cyclophosphamide-induced toxicity through the significant increase in the renal levels of glutathione (GSH) and superoxide dismutase (SOD) and the reduction in lipid peroxidation when compared to the control group [6]. Similarly, Husna et al. [7] observed that the ethanolic extract of curry leaves induced an antihyperglycemic effect in nicotinamide–streptozotocin-induced diabetic rats by decreasing the malondialdehyde level, associated with the generation of free radicals, and increasing the GSH level. Nevertheless, these and other potential pharmacological activities of *M. koenigii* extracts are principally attributed to their secondary metabolites such as alkaloids, terpenoids, and flavonoids, especially their major constituents, the carbazole alkaloids [8]. Currently, more than 80 distinct carbazole alkaloids from different parts of *M. koenigii* have been reported, such as mahanine, mahanimbine, koenimbine, girinimbine, and murrayafoline. [9]. Intriguingly, the most studied mahanine showed significant anticancer effects on glioma HS 683 cells by induction of apoptosis through the upregulation of Bax, cytochrome c, cleaved caspase 3 and 9, and cleaved poly (ADP-ribose) polymerase (PARP) [10]. Moreover, it was noticed that mahanine caused cell cycle arrest in glioma cancerous cells by the downregulation of M-phase inducer phosphatase 3 (Cdc25c), cell division cycle protein 2 homolog (Cdc2), and cyclin B1 [10].

MiRNAs are short (21–24 nucleotides), noncoding RNA molecules involved in the post-transcriptional regulation of gene expression in eukaryotes [11,12,13]. They are synthesized by RNA polymerase II as a primary transcript (pri-miRNA), which is subsequently processed by the DICER-LIKE1 (DCL1) protein to become the mature miRNAs. The mature miRNAs are loaded onto the ARGONAUTE proteins to form RNA-induced silencing complexes (RISCs) and bind complementarily to their corresponding mRNA target for either its degradation or translation inhibition [14]. Plant miRNAs not only serve as the master regulators of growth and development, but also are involved in many biological processes such as the control of phenotypic plasticity, biotic and abiotic stress responses, symbiotic and parasitism processes, and secondary metabolism [11,15,16,17,18]. Previously, Gou et al. [19] noticed that in *Arabidopsis thaliana*, miR156 targeted the transcription factor squamosa promoter binding protein-like 9 (SPL 9), which regulates the metabolic flux in the flavonoid biosynthetic pathway [19]. Likewise, in the medicinal herb *Picrorhiza kurroa*, miR4995 was found to target the mRNA of 3-deoxy-7-phosphoheptulonate synthase, an enzyme involved in the picroside biosynthetic pathway, a relevant compound known to protect from the ischemia/reperfusion injury [20,21]. In addition, in *Papaver somniferum*, miR2161 and miR13 were reported to target the transcripts of the enzymes 3′-hydroxy-N-methylcoclaurine 4′-O-methyltransferase 2 and 7-O-methyltransferase, implicated in the benzylisoquinoline alkaloid biosynthesis pathway [22]. Additionally, miR5140, miR159, miR477, and miR530 were found to be implicated in the biosynthesis regulation of withanolides, a group of compounds with significant medicinal properties such as anti-inflammatory and immunomodulatory, produced by *Withania somnifera* [23]. Moreover, in soybean (*Glycine max* L.), miR159, miR1534, and miR5030 regulate several transcription factors, including MYB65, MYB96, and MYB176, associated with isoflavonoid biosynthesis, such as chalcone synthase, chalcone isomerase, and isoflavone reductase [24]. Recently, Mishra et al. [25] reported that in economically important perennial bunchgrass *Chrysopogon zizanioides* (L.) Roberty (primarily used in the perfumery industry), miR2102, miR854, and miR5658 targeted enzymes that are involved in the terpenoid biosynthesis pathway.

Due to a significant association of several miRNAs in plant secondary metabolite biosynthesis, plant biotechnologists recently have begun to exploit and/or manipulate different relevant microRNAs as a useful tool for controlling secondary metabolites biosynthetic pathways not only in model plants, but also in economically/medicinally important ones [26,27,28,29]. For example, an artificial inhibition of Sm-miR408 expression in the traditional herb *Salvia miltiorrhiza* was found to enhance the root accumulation of salvianolic acid B, a chemical compound that has hepatoprotective, cardioprotective, and anticancer properties [30]. Similarly, the production of the commercially used plant-derived sweet compounds steviol glycosides from *Stevia rebaudiana* was increased 24.5 and 51% by anti-miR319g and miRStv_11 coexpression, which target genes related to its biosynthetic pathway [31]. Furthermore, the synthesis of guaianolides, byproducts of chicory (*Cichorium intybus* L.), was reduced by an artificial miRNA that targeted germacrene A synthase genes, aiming to improve the extraction of inulin, a commercial compound used in the food industry as a prebiotic and sweetener [32].

Currently, a complete miRNA profile and their expression pattern can be investigated authentically using next-generation sequencing (NGS) technology in medicinal plants [33,34,35], which in turn allows elucidating the functions of their potential target transcripts and their relationship with secondary metabolites biosynthesis, strengthening transgenic research. It is also well established that secondary metabolite production can be enhanced in planta by manipulating target transcripts with artificial miRNAs (amiRNAs). However, in order to exploit them in the metabolic engineering process, a better understanding of their modes of action is required. Thus, the present study aimed to generate for the first time an NGS-based miRNA profile of *M. koenigii,* and to predict their potential targets associated with the secondary metabolism biosynthesis pathways.

## 2. Results

### 2.1. Sequence Analysis of M. koenigii Small RNAs

**Figure 1:**
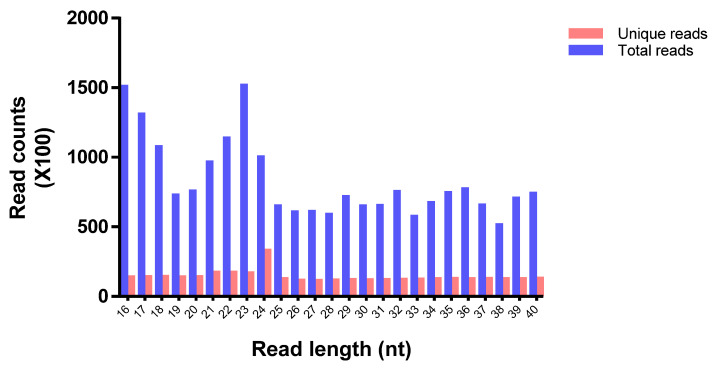
Length distribution and abundance of the small RNA sequences identified in *M. koenigii* leaves through Illumina sequencing.

In this study, utilizing high-throughput Illumina sequencing technology, a total of 8,186,145 raw reads were obtained from the *M. koenigii* leaf tissue samples. The raw data of small RNA sequencing were uploaded to the NCBI SRA database (Accession number: SRR16796893). After removing the adaptors, low-quality reads, and other small RNAs such as rRNA (156,700), snoRNA (1483), snRNA (1315), and tRNA (18) (Table 1), a total of 343,505 unique reads with lengths ranging from 16 to 40 nt were attained. The size distribution of unique *M. koenigii* reads showed that 24 nt represented the most abundant one (9.52%) followed by 21 nt (4.94%), 22 nt (4.92%), and 23 nt (4.79%) (Figure 1).

### 2.2. Identification of Conserved and Novel miRNAs in M. koenigii

**Figure 2:**
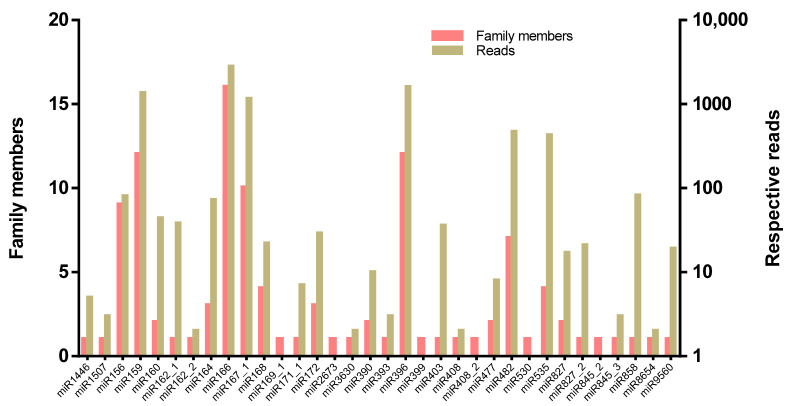
Family members and their respective reads within each miRNA family of *M. koenigii*.

To identify conserved miRNAs in *M. koenigii*, the unique reads were aligned against miRbase-22 using the BLASTn tool, and a total of 142 conserved miRNAs, representing 34 miRNA families, were identified (Table 2). All the conserved miRNAs showed significant homology (no more than one mismatch) with their respective homologs, and their frequency varied widely between families. The most abundant miRNA families were miR166, miR159, miR396, and miR167 with 16, 12, 12, and 10 members, respectively (Figure 2). The read counts of the miRNA families varied from 1 to 2795, where the miR166 family had the highest number of reads (2795), followed by the miR396 (1598) and miR159 (1364) families.

**Figure 3:**
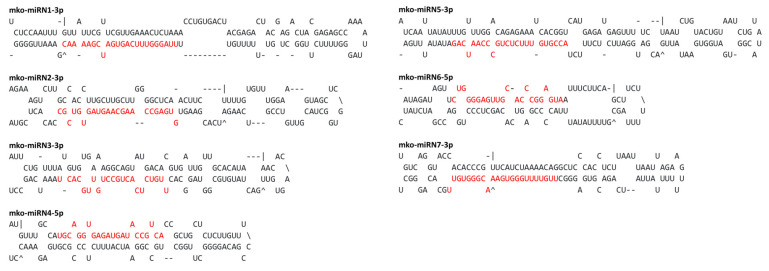
Secondary structures (stem-loops) of *M. koenigii* novel miRNA precursors. Mature miRNAs are highlighted in red font.

Following the identification of conserved miRNAs, the remaining unaligned 42,658 reads were subjected to predict novel *M. koenigii* miRNA candidates by applying strict filtering criteria. A total of seven novel miRNAs were identified in this study (Table 3), and their secondary hairpin structures were deduced (Figure 3). The read counts of these novel miRNA candidates varied from 5 to 93. The mko-miRN7-3p displayed the highest number of reads (93), followed by mko-miRN4-5p (15) and mko-miRN1-3p (14). The precursor sequences of these novel miRNA candidates had high Minimum Folding Free Energy Index (MFEI) values ranging from 0.70 to 0.98 with an average of 0.85 ± 0.11, distinguishing them from other types of RNAs such as tRNAs (0.64), rRNAs (0.59), and mRNAs (0.62–0.66) [36].

### 2.3. Target Prediction of Conserved and Novel M. koenigii miRNAs and Their Functional Analysis

**Figure 4:**
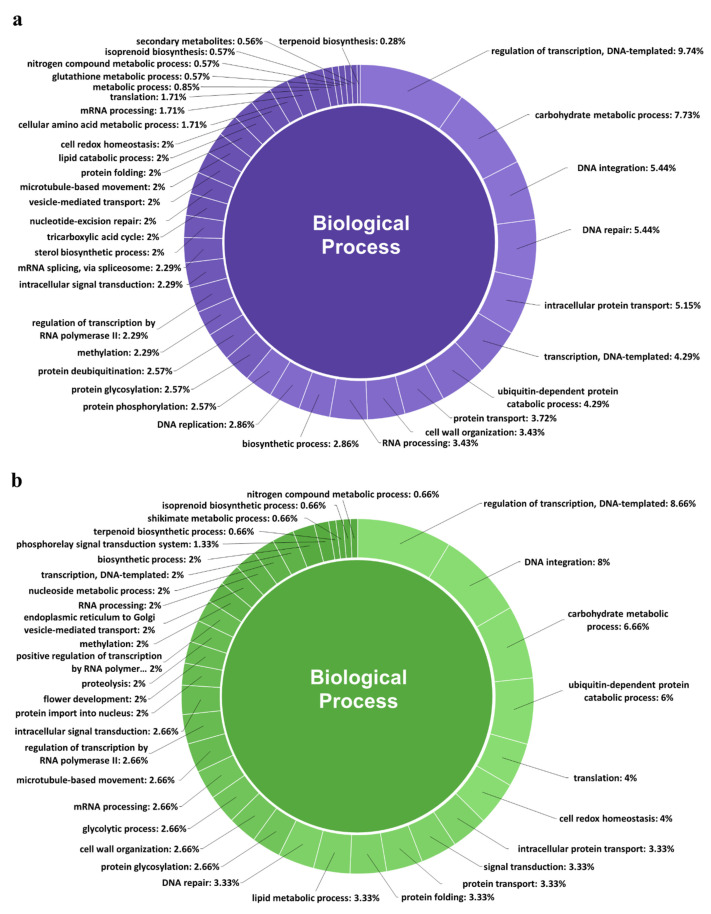
Biological Process categories of the GO analysis of the potential target genes of both conserved (**a**) and novel (**b**) miRNAs from *M. koenigii*.

**Figure 5:**
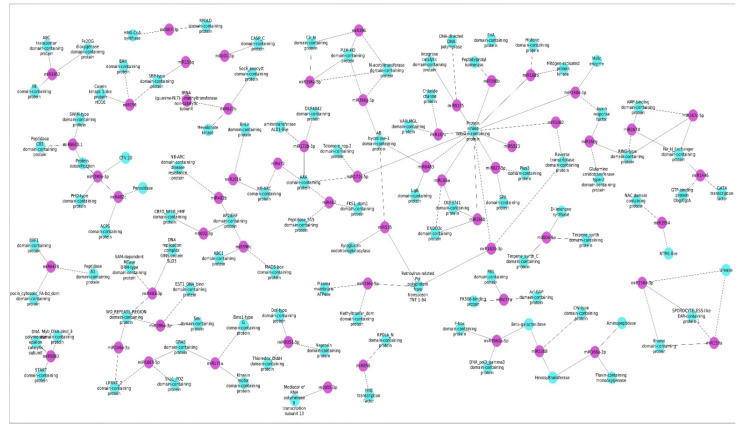
MFE-based network interaction of *M. koenigii* miRNAs and their corresponding potential targets. Coregulation of multiple targets was observed for several of the identified miRNAs (weaker energy interactions were marked as dotted lines).

In this study, a total of 6078 corresponding potential target genes of *M. koenigii* miRNAs were identified (5196 target genes for conserved miRNAs and 882 target genes for novel miRNAs). A total of 83% of the target genes of the conserved miRNAs were found to be regulated by direct cleavage of transcripts, while the remaining targets were by translational repression, whereas 100% of the target genes of the novel miRNAs were regulated by direct cleavage. In the case of conserved miRNAs, mko-miR827-5p (221) had the highest number of potential targets, followed by mko-miR396c (214) and mko-miR396a-3p (203); while for novel miRNAs, mko-miRN7-3p (196) targeted the greatest number of transcripts. Moreover, the Gene Ontology (GO) analysis revealed that for the targets of conserved miRNAs, the main terms in the Biological Process (BP) category were “regulation of transcription, DNA-templated” (9.74%), “carbohydrate metabolic process” (7.73%), and “DNA integration” (5.44%); however, “nitrogen compound metabolic process” (0.57%), “isoprenoid biosynthesis” (0.57%), “secondary metabolites” (0.56%), and “terpenoid biosynthesis” (0.28%) were key terms related to secondary metabolism biosynthesis. Furthermore, regarding the targets of novel miRNAs in the BP category, the principal terms were “regulation of transcription, DNA-templated” (8.66%), “DNA integration” (8%), and “carbohydrate metabolic process” (6.66%), and specifically the “terpenoid biosynthetic process” (0.66%), “shikimate metabolic process” (0.66%), “isoprenoid biosynthetic process” (0.66%), and “nitrogen compound metabolic process” (0.66%) terms were involved in the biosynthesis of secondary metabolites (Figure 4). Furthermore, gene network analysis revealed coregulation of numerous target genes (Figure 5).

### 2.4. Human Target Gene Prediction of M. koenigii miRNAs

Apart from regulating the secondary metabolism and other biological processes, plant miRNAs are also known to regulate human target genes with the potential for treating human diseases [37,38,39]. In this study, the human target prediction analysis indicated that 48 conserved *M. koenigii* miRNAs targeted 4362 human genes. Specifically, mko-miR160g, mko-miR160e-5p, and mko-miR477 accounted for the highest number of human targets: 516, 476, and 384, respectively. Interestingly, several *M. koenigii* miRNAs such as mko-miR5082, mko-miR5368, mko-miR482b, mko-miR156q, and mko-miR396a-5p targeted proteins involved in pathways related to inflammation, cancer, and neurological disorders including Alzheimer’s, Parkinson’s, and Huntington’s. Regarding the novel miRNAs of *M. koenigii*, mko-miRN6-5p targeted the highest number (161) of human genes, followed by mko-miRN3-3p (75) and mko-miRN2-3p (62), which are related to insulin secretion, cardiomyopathy, and autoimmune thyroid disease pathways, respectively. Nevertheless, the precise implications of *M. koenigii* miRNAs in human health must be elucidated further with relevant experimental approaches.

### 2.5. Identification of M. koenigii miRNA Targets Involved in Plant Secondary Metabolite Biosynthesis

**Figure 6:**
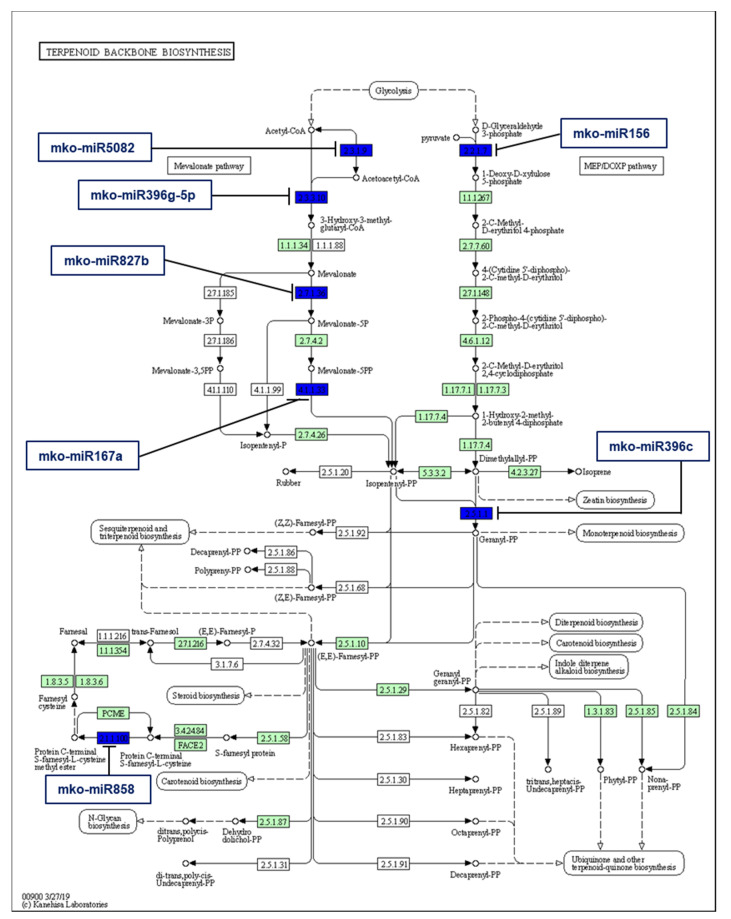
Target enzymes of conserved miRNAs of *M. koenigii* in the terpenoid backbone biosynthesis pathway. EC:2.2.1.7—1-deoxy-D-xylulose-5-phosphate synthase; EC:2.3.1.9—acetyl-CoA C-acetyltransferase; EC:4.1.1.33—diphosphomevalonate decarboxylase; EC:2.1.1.100—protein-S-isoprenylcysteine O-methyltransferase; EC:2.5.1.1—geranyl diphosphate synthase; EC:2.3.3.10—hydroxymethylglutaryl-CoA synthase; EC:2.7.1.36—mevalonate kinase. The blue boxes represent the targeted enzymes of the corresponding known miRNAs.

The medicinal properties of *M. koenigii* can be attributed to the presence of secondary metabolites synthesized by specific enzymes. Therefore, in this study, our principal goal was to identify the *M. koenigii* miRNAs whose target genes coded for enzymes involved in the secondary metabolite biosynthesis. The results revealed a total of 286 target genes of *M. koenigii* miRNAs associated with ubiquinone and terpenoid-quinone biosynthesis; tropane, piperidine, and pyridine alkaloid biosynthesis; isoquinoline alkaloid, sesquiterpenoid, and triterpenoid biosynthesis; terpenoid backbone biosynthesis; mevalonate biosynthesis; diterpenoid biosynthesis; flavonoid biosynthesis; flavone and flavonol biosynthesis; and isoprenoid biosynthesis. In addition, seven *M. koenigii* miRNAs (mko-miR156, mko-miR5082, mko-miR167a, mko-miR858, mko-miR396c, mko-miR396g-5p, and mko-miR827b) controlled the key enzymes (1-deoxy-D-xylulose-5-phosphate synthase, acetyl-CoA C-acetyltransferase, diphosphomevalonate decarboxylase, protein-S-isoprenylcysteine O-methyltransferase, geranyl diphosphate synthase, hydroxymethylglutaryl-CoA synthase, and mevalonate kinase) that regulate the terpene backbone pathway (Figure 6), which allows structural diversity to give rise to many types of terpenoids such as monoterpenoids, sesquiterpenes, and diterpenes.

**Figure 7:**
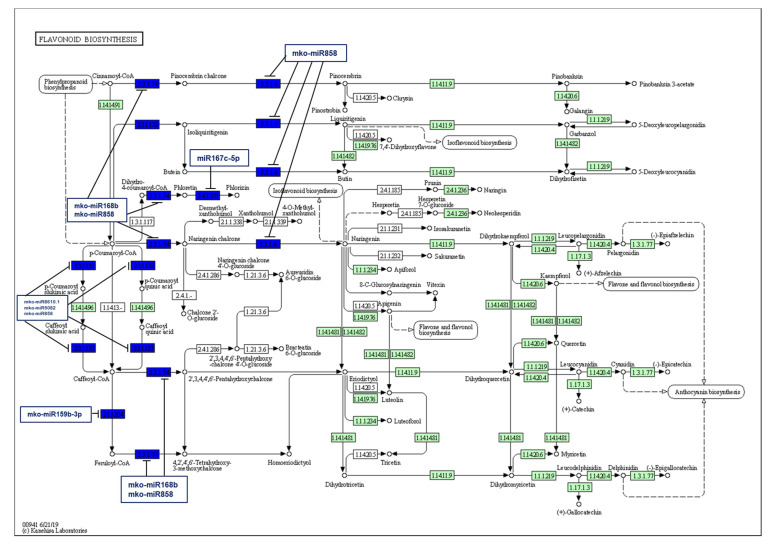
Target enzymes of conserved miRNAs of *M. koenigii* in the flavonoid biosynthesis pathway. EC:2.3.1.74—chalcone synthase; EC:5.5.1.6—chalcone isomerase; EC:2.1.1.104—caffeoyl-CoA O-methyltransferase; EC:2.3.1.133—shikimate O-hydroxycinnamoyltransferase; EC:2.4.1.357—phlorizin synthase. The blue boxes represent the targeted enzymes of the corresponding known miRNAs.

Furthermore, this study revealed that chalcone synthase, which plays a pivotal role in the flavonoid biosynthesis pathway, was targeted by mko-miR168b and mko-miR858; while chalcone isomerase, another important enzyme in the flavonoid synthesis pathway, was also targeted by mko-miR858 only. Additionally, mko-miR858, mko-miR8610.1, and mko-miR5082 targeted shikimate O-hydroxycinnamoyltransferase, an enzyme that produces quinate or shikimate ester using p-coumaroyl CoA as an acyl donor. In a subsequent reaction, this enzyme also was responsible for transferring the caffeoyl moiety of 5-O-caffeoylquinate/5-O-caffeoylshikimate onto coenzyme A, creating caffeoyl CoA, which in turn was methylated by the caffeoyl-CoA O-methyltransferase, an enzyme targeted by mko-miR159b-3p, for yielding feruloyl Co-A, an important precursor for the synthesis of anthocyanins and coumarins. Moreover, mko-miR167c-5p targeted phlorizin synthase, the enzyme that adds a glucose molecule to phloretin, resulting in phlorizin, a dihydrochalcone (Figure 7).

**Figure 8:**
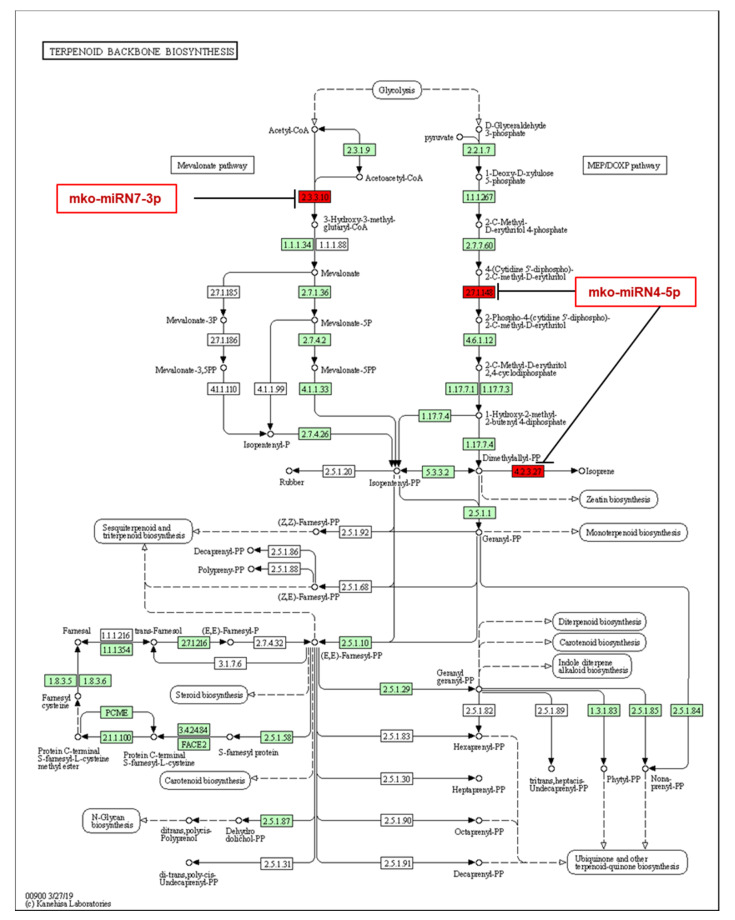
Target enzymes of the novel miRNAs of *M. koenigii* in the terpenoid backbone biosynthesis pathway. EC:4.2.3.27—isoprene synthase; EC:2.7.1.148—4-diphosphocytidyl-2-C-methyl-D-erythritol kinase; EC:2.3.3.10—hydroxymethylglutaryl-CoA synthase. The red boxes represent the targeted enzymes of the corresponding novel miRNAs.

Interestingly, several targets of *M. koenigii* novel miRNAs were also implicated in several secondary metabolite biosynthetic pathways, including terpenoid backbone biosynthesis; isoquinoline alkaloid biosynthesis; betalain biosynthesis; diterpenoid biosynthesis; indole alkaloid biosynthesis; monoterpenoid biosynthesis; mevalonate biosynthesis; sesquiterpenoid and triterpenoid biosynthesis; tropane, piperidine, and pyridine alkaloid biosynthesis; and ubiquinone and other terpenoid-quinone types of biosynthesis. For example, mko-miRN4-5p targeted both isoprene synthase and 4-diphosphocytidyl-2-C-methyl-D-erythritol kinase genes, which belong to the mevalonate-independent pathway; whereas mko-miRN7-3p targeted hydroxymethylglutaryl-CoA synthase, a crucial enzyme for the downstream synthesis of mevalonate (Figure 8).

### 2.6. Experimental Validation of M. koenigii miRNAs by qPCR

**Figure 9:**
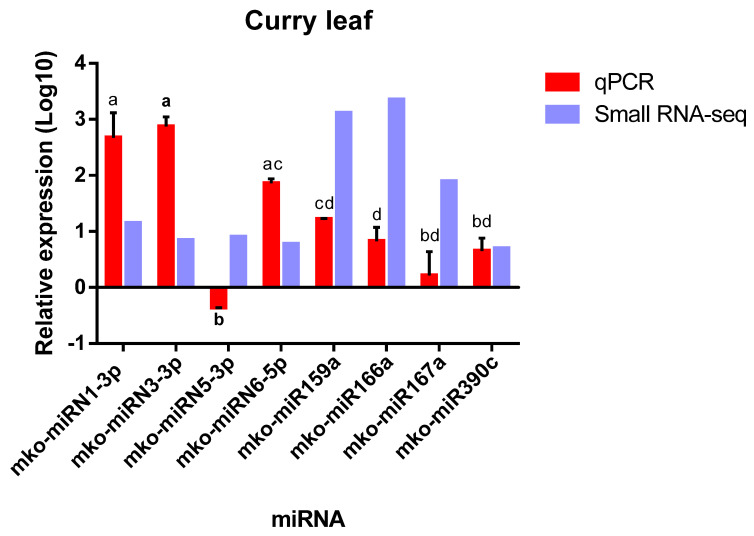
Quantitative PCR analysis of conserved and novel miRNAs of *M. koenigii*. The relative expression (Log10) of 4 conserved and 4 novel miRNAs was detected by qPCR. U6 was used as an endogenous control. The expression level of miR156a-5p was set as control and taken as 1, and the expression level in all other miRNAs was quantified relative to it. The analysis was performed as triplicates, and the error bars indicate standard deviations. Significant differences between miRNAs at *p* < 0.05 by ANOVA and Tukey’s test are indicated with different letters.

Four conserved and four novel *M. koenigii* miRNAs were randomly selected for qPCR analysis to validate the high-throughput sequencing data. The results showed similar expression patterns between the Illumina sequencing and qPCR analysis, except for mko- miRN5-3p. Moreover, the post hoc statistical analysis of the qPCR data indicated that the relative expression of most of the validated miRNAs was similar (as specified by the letters above the bars in Figure 9).

## 3. Discussion

Plant secondary metabolites are molecules synthesized under specific situations to interact with the environment and to adapt to biotic and abiotic stress conditions [40]. Their biosynthesis is highly energy-consuming, making it a tightly regulated process [11]. Few studies have demonstrated the important regulatory roles of miRNAs in plant secondary metabolism [11,41]. In the present work, the Illumina small-RNA sequencing technology was employed to explore the miRNA profile of the medicinal plant *M. koenigii*, aiming to understand their implication in secondary metabolism.

It has been stated that miRNAs can either target the transcripts of the enzymes responsible for the biosynthesis of these secondary metabolites or the transcripts of regulatory proteins that control the expression of the former, such as transcription factors [42]. In this study, the conserved *M. koenigii* miRNAs were distributed in 34 families, with miR166 as the family with the highest number of members (16) and the highest number of total reads (2795) compared to other families. Kajal and Singh [43] reported that miR166i-3p was involved in regulating sesquiterpenes and triterpenoids targeting the squalene synthase in *Chlorophytum borivilianum*, which produces a type of saponins known as borivilianosides, with several pharmacological activities such as immunomodulatory, antidiabetic, and androgenic. However, in contrast, it has been reported that the miR166 family from blueberry (*Vaccinium ashei*) can target the transcription factor squamosa promoter binding protein-like (SPL), which prevents the expression of the biosynthetic genes of anthocyanins flavonoid [42].

Several studies investigated the essential oil composition from the leaves of *M. koenigii*, which varies depending on the type of oil extraction and the sample origin [44,45]. Nevertheless, they coincided in that it contained C10 monoterpenes and C15 sesquiterpenes. For example, Verma et al. [46] reported that α-pinene, sabinene, (E)-caryophyllene, β-pinene, terpinen-4-ol, γ-terpinene, limonene, α-terpinene, (E)-nerolidol, α-humulene, α-thujene, and β-elemene were the major components in the essential oil of *M. koenigii* from the Western Himalayas. The backbone structure of these terpenoids was built up with C5 isoprenoid units. Intriguingly, the present study showed that both conserved and novel *M. koenigii* miRNAs were implicated in the terpenoid backbone biosynthesis, which can be synthesized either by the mevalonate pathway (MVA) in the cytoplasm or by the 2C-methyl-d-erythritol-4-phosphate pathway (MEP) in the plastids [47]. The former pathway focuses on synthesizing monoterpenes, diterpenes, and tetraterpenes, while the latter focuses on the sesquiterpenes, sterols, and triterpenes [48]. In this context, the target prediction analysis of *M. koenigii* miRNAs revealed that mko-miR5082 targeted the enzyme acetyl-CoA C-acetyltransferase, responsible for condensing two molecules of acetyl-CoA into acetoacetyl-CoA, a very first step in terpenoid backbone synthesis; while mko-miR396g-5p and the novel mko-miRN7-3p targeted the enzyme hydroxymethylglutaryl-CoA synthase, which condenses the third acetyl-CoA into 3-hydroxy-3-methylglutaryl-CoA. A similar kind of work has also been reported in *Rauvolfia serpentina,* where miR396 targeted the secologanin synthase, an oxidoreductase involved in synthesizing the monoterpene secologanin [49]. Additionally, mko-miR827b targeted the mevalonate kinase, whereas mko-miR167a targeted the mevalonate diphosphate decarboxylase, two important enzymes acting sequentially and responsible for supplying the C5 prenyl diphosphates for the downstream terpenoid biosynthesis. It was also found that mko-mir156 targeted 1-Deoxy-d-xylulose 5-phosphate synthase, which performs a committed step in the MEP pathway, controlling its flux. Our results corroborated those of Singh et al. [50], who claimed that miR156 targeted the same aforesaid enzyme in *Mentha* spp. Likewise, another study showed that miR156 targeted SLP9, a transcription factor that modulates the expression of the sesquiterpene synthase gene TPS21 in *Pogostemon cablin* (patchouli) [51]. Furthermore, mko-mir396c targeted geranyl diphosphate synthase, the principal enzyme responsible for producing geranyl diphosphate, a crucial molecule for monoterpenoid and diterpenoid biosynthesis [48]. Regarding the novel *M. koenigii* miRNAs, mko-miRN4-5p targeted both the enzyme 4-diphosphocytidyl-2-C-methyl-D-erythritol kinase and the isoprene synthase, with the latter producing isoprene from dimethylallyl diphosphate in the MEP pathway.

Several well-known flavonoids such as rutin, quercetin, myricetin, kaempferol, and catechin have already been extracted from *M. koenigii* leaves [52,53]. These kinds of molecules are initially synthesized through the phenylpropanoid pathway, in which phenylalanine is first converted into 4-coumaroyl-CoA and then enters into the flavonoid biosynthesis pathway [54]. The current study revealed that the conserved mko-miR168b, mko-miR858, mko-miR8610.1, and mko-miR5082 targeted three important enzymes: chalcone synthase, chalcone isomerase, and shikimate O-hydroxycinnamoyltransferase, associated with the flavonoid biosynthesis pathway. Specifically, chalcone synthase produces, among other chalcones, naringenin chalcone and pinocembrin chalcone, which in turn are converted into naringenin and pinocembrin by chalcone isomerase. Similarly, in blueberry, chalcone synthase is targeted by miR166 and miR390 families, whereas flavonol synthase is targeted by the miR159, miR171_1, and miR845_1 families [42]. Additionally, studies of *A. thaliana* and *Diospyros kaki* showed that miR858 targeted MYB transcription factors, which regulate genes of the flavonoid and proanthocyanidin biosynthesis [55,56]. In addition, miR159b-3p targeted caffeoyl-CoA O-methyltransferase, the enzyme responsible for synthesizing feruloyl-CoA from caffeoyl-CoA; and miR167c-5p targeted phlorizin synthase, which converts phloretin into phlorizin, its glucoside form. Nevertheless, further studies are needed to explain the potential role of these enzymes in the flavonoid biosynthesis pathway of *M. koenigii* leaves.

The validation analysis was performed for four novel and four conserved *M. koenigii* miRNAs. It revealed similar expression patterns between the qPCR experiments and the Illumina sequencing. This behavior was also observed for blueberry miRNAs and ramie (*Boehmeria nivea*) miRNAs [42,57]. However, contrary to the sequencing result, the qPCR data showed that the novel miRNA mko-miRN5-3p resulted in negative relative expression. This could be attributed to either low-quality primers or probable low abundance of the miRNA, as previously reported in *G. max* (miR393a), *Elettaria cardamomum* (miR477e), and *Catharanthus roseus* (cro-novel-71, cro-novel-98, cro-novel-43, cro-novel-38, and cro-novel-58) [58,59,60].

There is growing evidence of the cross-kingdom miRNA transfer between humans and plants [37]. Moreover, studies suggested that plant miRNAs have the potential to treat human diseases [38,39]. For example, it was reported that miR159 not only inhibited the cell proliferation of the breast cancer cell line MDA-MB-231, but also suppressed the growth of mice xenograft breast tumors [61]. Likewise, Li et al. [62] observed that miR167e-5p inhibited the proliferation of enterocytes (IPEC-J2) and human colon carcinoma (Caco-2) cell lines in vitro. The present study found that several *M. koenigii* miRNAs, including mko-miR8175, mko-miR5368, and mko-miR156, targeted genes encoding proteins involved in colorectal, prostate, and breast cancers, corroborating the report of Xie et al. [63], who showed that val-miR1086 and val-miR1127 from *Viscum album* (mistletoe) could regulate the expression level of the p53 tumor suppressor gene. In this context, the predicted human targets of *M. koenigii* might shed light on its pharmacological properties. Nevertheless, further experimental validation is needed.

## 4. Materials and Methods

### 4.1. Plant Materials and RNA Extraction

Leaf samples from healthy 5-year-old-M. *koenigii* plants (grown under natural conditions) were collected, instantly frozen in liquid nitrogen, and subsequently stored at −80 °C until used. Total RNA was isolated from 100 mg of leaf sample using a Spectrum™ Plant Total RNA Kit (Sigma-Aldrich, St. Louis, MO, USA) following the manufacturer’s instructions. The quality and quantity of the RNA were assessed using Nanodrop2000 (Thermo Scientific, Wilmington, DE, USA), Qubit (Thermo Scientific, Wilmington, DE, USA), and Bioanalyzer 2100 (Agilent, Palo Alto, CA, USA).

### 4.2. Small RNA Library Construction and Sequencing

The small RNA library was constructed using the QIAseq^®^ miRNA Library Kit (Qiagen, Germantown, MD, USA) protocol. Briefly, 100 ng of total RNA was utilized as initial material, and 3′ adapters were ligated to the specific 3′OH group of microRNAs followed by 5′ adapters ligation. Consequently, the adapter-ligated fragments were reverse transcribed with Unique Molecular Index (UMI) assignment, and the cDNA was barcoded and amplified in a single step by PCR. The resulting cDNA library was then quantified by Qubit fluorometer (Thermo Fisher Scientific, Waltham, MA, USA), and the fragment size distribution was analyzed with an Agilent 2200 Tapestation system. Finally, the sequencing was performed for 75 cycles on an Illumina NextSeq 550 High Output sequencing platform following the manufacturer’s protocol.

### 4.3. Small RNA Sequencing Data Analysis

Following the completion of the sequencing run, the Illumina GA raw data were processed for the removal of adaptors and low-quality reads (<Q30) using sRNA-workbench (V3.0_ALPHA). Sequences smaller than 16 bp and larger than 40 bp, as well as reads matching other ncRNAs (rRNA, tRNA, snRNA, and snoRNAs), were eliminated. The remaining small RNA sequences were aligned against miRbase-22.1 (http://www.mirbase.org, assessed on 10 June 2021) to identify conserved *M. koenigii* miRNAs. Subsequently, sequences not showing homology were considered for the prediction of novel miRNAs using bowtie [64] and Mireap_0.22b [65]. However, due to the unavailability of the *M. koenigii* genome, the well-annotated *Citrus sinensis* (belonging to the same family of *M. koenigii*) genome was used as the reference. The novel miRNAs with proper precursor secondary structures and MFEI values of ≥0.70 were only considered for this study. The secondary structures of the precursors were predicted using the UNAFold Web Server (http://www.unafold.org, assessed on 10 June 2021), and the MFEI values were calculated as follows:

$$\mathrm{MFEI}=\frac{\left(\mathrm{MFE}/\mathrm{length}\;\mathrm{of}\;\mathrm{RNA}\;\mathrm{sequence}\right)\times 100}{\%\;\mathrm{GC}\;\mathrm{content}}$$

### 4.4. Prediction of M. koenigii miRNA Targets, Their Functional Annotation, and Pathway Analysis

The conserved and novel miRNAs with copy numbers more than equal to 5 were considered for target gene prediction using the psRNATarget tool (https://www.zhaolab.org/psRNATarget, assessed on 10 June 2021). For human target prediction, the miRanda tool was used, in which the miRNA sequences were analyzed along with the *human cDNA list* in strict mode (strict alignment of seed regions), and miRNA hits having minimum free energy ≥ −25 were assumed to be potential targets. GO annotation of the potential *M. koenigii* miRNAs targets was performed in the BP category (considering top 30 and secondary metabolism associated terms). Moreover, to find the coregulation of the potential targets, a biological network was generated using the MFE values of the miRNA–target interaction, and the biological network of the miRNAs and their targets was visualized using Cytoscape 3.2 (https://cytoscape.org/release_notes_3_2_0.html, assessed on 10 June 2021). Finally, miRNA targets associated with the secondary metabolite biosynthetic pathways were analyzed precisely.

### 4.5. Extraction of Small RNA and Experimental Validation of M. koenigii miRNAs by qPCR

To validate the identified conserved and novel miRNA candidates, small RNAs were isolated from a frozen leaf sample of *M. koenigii* using the mirVana™ miRNA Isolation Kit (Thermo Fisher Scientific, Waltham, MA, USA) following the manufacturer’s instructions. The quality and quantity of the small RNA samples were measured with a NanoDrop One UV–Vis microvolume spectrophotometer (Thermo Scientific™, Wilmington, DE, USA). Following the quality check, small RNAs were polyadenylated and reverse transcribed using the Mir-X miRNA First-Strand Synthesis kit (Clontech, Mountain View, CA, USA), and finally, the qPCR was performed using the TB Green^®^ Advantage^®^ qPCR Premix (Takara Bio USA, Inc., San José, CA, USA) in a StepOne™ Real-Time PCR System (Applied Biosystems, Carlsbad, CA, USA). The reactions were performed in a 48-well optical plate using the following conditions: an initial polymerase activation step for 10 s at 95 °C, followed by 45 cycles of 5 s at 95 °C for denaturation, and 20 s at 55 °C for annealing and extension. The amplification cycle was followed by a melting curve analysis ranging from 56–95 °C, with temperature increases in steps of 0.5 °C every 10 s. The reactions were performed with two biological replicates and three technical replicates for each sample, and the relative expression of the miRNAs was quantified by the 2^−ΔΔCt^ method utilizing U6 as endogenous control. Additionally, the expression level of miR156a-5p was set as control (taken as 1), and all other miRNA expression was quantified relative to it.

## 5. Conclusions

In this study, a total of 142 conserved and 7 novel miRNAs from *M. koenigii* were identified using high throughput sequencing technology. Among the identified miRNAs, seven conserved and two novel miRNAs were found to target enzymes significantly involved in the terpenoid backbone biosynthesis pathway, while six conserved miRNAs were found to target enzymes of the flavonoid biosynthesis pathway. Moreover, the human target prediction analysis revealed that *M. koenigii* miRNAs potentially targeted genes implicated in human health. Finally, eight miRNAs were experimentally validated using qPCR. Notably, this is the first report of microRNAs from the medicinal plant *M. koenigii* and their association with secondary metabolite biosynthesis. This study might strengthen the miRNA-mediated transgenic research for the overproduction of medicinally as well as commercially valuable plant secondary metabolites.

## Figures and Tables

**Table 1:** Categorization of sequencing reads.

Category	Total Reads	Unique Reads
Total reads	8186145	3726171
Trimmed reads	2052756	343505
% reads aligned to ncRNA	46.44%	
Reads aligned to ncRNA (rRNA, snoRNA, snRNA, tRNA)	1,268,546	159,516
Reads aligned to miRBase	15,388	556
Known miRNA	142	
Reads used for novel miRNA	41,879	23,286
Novel miRNA	7	
Putative miRNA	726,943	79,115

**Table 2:** Summary of *M. koenigii* conserved miRNAs.

miRNA Family	Name	Sequence (5’-3’)	Length (nt)	Reference miRNA	No. of Mismatches	Read Counts	E Value
MIR156	mko-miR156	CTGACAGAAGAGAGTGAGCAC	21	ama-miR156	0	17	0.0000002
	mko-miR156	TTGACGGAAGATAGAGAGCAC	22	bgy-miR156	1	6	0.00003
	mko-miR156a	TGACAGAAGAGAGTGAACACA	21	bna-miR156a	1	2	0.00004
	mko-miR156a-3p	GCTCACTGCTCTTTCTGTCAG	22	ath-miR156a-3p	0	7	0.0000001
	mko-miR156d-3p	GCTCTCTATGCTTCTGTCATCA	22	stu-miR156d-3p	0	1	0.00000004
	mko-miR156e-5p	TGATAGAAGAGAGTGAGCA	20	sly-miR156e-5p	0	1	0.000002
	mko-miR156f-3p	TGCTCACTGCTCTTTCTG	23	bra-miR156f-3p	0	4	0.000009
	mko-miR156q	TGACAGAAGAGAGTGAGCACT	21	gma-miR156q	0	39	0.0000002
	mko-miR157d-3p	GCTCTCTATTCTTCTGTCATC	21	aly-miR157d-3p	1	3	0.00004
MIR159	mko-miR159	TTTGGGTTGAAGGGAGCTCTA	21	pde-miR159	1	2	0.00004
	mko-miR159	TTTGGACTGAAGGGAGCTCTA	21	aqc-miR159	0	1	0.0000002
	mko-miR159a	TTTGGATTGAAGGGAGCTCTA	21	ath-miR159a	0	1296	0.0000003
	mko-miR159a-5p	GAGCTCCTTGAAGTCCAA	21	gma-miR159a-5p	0	21	0.000009
	mko-miR159b	TTGCATATCTCTGGAGCTTC	21	hbr-miR159b	1	1	0.0001
	mko-miR159b-3p	TTTGGATTGAAGGGAGCTCTT	21	ath-miR159b-3p	0	29	0.0000002
	mko-miR159c	TTTGGATTGAAGGGAGCTC	21	ath-miR159c	0	4	0.000002
	mko-miR159c	ATTGGATTGAAGGGAGCTC	21	osa-miR159c	0	1	0.000002
	mko-miR159c-5p	TTGGATCGAAGGGAGCTC	21	zma-miR159c-5p	0	3	0.000009
	mko-miR159d	AGCTGCTGAGCTATGGATCCC	21	gma-miR159d	1	4	0.00009
	mko-miR159e	TTTGGATTGAAAGGAGCTCT	21	sof-miR159e	0	1	0.0000006
	mko-miR159f	TTGGATTGAACGGAGCTCTA	21	osa-miR159f	1	1	0.0001
MIR160	mko-miR160e-5p	TGCCTGGCTCCCTGTATGCCG	21	osa-miR160e-5p	0	14	0.0000002
	mko-miR160g	GCCTGGCTCCCTGTATGCCA	23	mes-miR160g	0	30	0.0000009
MIR162_1	mko-miR162b-3p	CGATAAACCTCTGCATCCAG	21	ath-miR162b-3p	0	38	0.0000006
MIR162_2	mko-miR162b	TCGATAAGCCTCTGCATCCAG	21	osa-miR162b	0	2	0.0000002
MIR164	mko-miR164b	TGGAGAAGCAGGGCACGT	20	gma-miR164b	0	68	0.000009
	mko-miR164b-5p	TGGAGAAGTAGGGCACGTGCA	21	ath-miR164b-5p	1	2	0.00004
	mko-miR164d	TGGAGAAGCAGGGCACATGCT	21	mtr-miR164d	0	2	0.0000002
MIR166	mko-miR165b	TCGGACCAGGCTTCATCCCC	21	ath-miR165b	0	2	0.0000006
	mko-miR166a	TCGGACCAGGCTTCATTCCCC	21	pta-miR166a	0	2258	0.0000003
	mko-miR166b	TCGGACCAGGCTTCATTCCCT	22	crt-miR166b	0	362	0.0000002
	mko-miR166b	TCGGACCAGGCTTCATTCCT	21	mtr-miR166b	0	5	0.0000006
	mko-miR166b	TCGGANCAGGCTTCATTCCCG	22	csi-miR166b	1	1	0.000009
	mko-miR166c-5p	GGAATGTTGTCTGGCTCGAGG	21	gma-miR166c-5p	0	144	0.0000003
	mko-miR166d	TCGGGCCAGGCTTCATTCCCC	21	mtr-miR166d	1	2	0.00004
	mko-miR166e-3p	TCGAACCAGGCTTCATTCCCC	21	osa-miR166e-3p	0	2	0.0000002
	mko-miR166h-5p	GGAATGTTGTTTGGCTCGAGG	21	gma-miR166h-5p	0	6	0.0000001
	mko-miR166i	TCGGACCAGGCTTCATTCT	20	cme-miR166i	0	2	0.000002
	mko-miR166i-3p	TCGGATCAGGCTTCATTCC	21	osa-miR166i-3p	0	1	0.000002
	mko-miR166k	TCTCGGACCAGGCTTCGTTCC	21	gma-miR166k	1	2	0.00004
	mko-miR166k-3p	CGGACCAGGCTTCAATCCC	21	osa-miR166k-3p	0	2	0.000002
	mko-miR166m	CGGACTAGGCTTCATTCCCC	20	gma-miR166m	1	1	0.0001
	mko-miR166p	TCGGACCAGGCTCCATTCC	21	ptc-miR166p	0	2	0.000002
	mko-miR166u	TCTCGGACCAGGCTTCATT	20	gma-miR166u	0	3	0.000002
MIR167_1	mko-miR167a	AGATCATCTGGCAGTTTCACC	21	mdm-miR167a	0	78	0.0000002
	mko-miR167b	TGAAGCTGACAGCATGATCT	21	tae-miR167b	0	2	0.0000006
	mko-miR167b	TGAAGCTGCCAGCATGATCTA	22	bna-miR167b	0	1	0.0000001
	mko-miR167c-5p	TGAAGCTGCCAGCATGATCTGC	22	tae-miR167c-5p	0	1047	0.00000004
	mko-miR167d	TGAAGCTGCCAGCATGATCTGA	22	cpa-miR167d	0	19	0.00000004
	mko-miR167d	TGAAGCAGCCAGCATGATCTGG	22	ath-miR167d	1	3	0.000009
	mko-miR167d	TGAAGCTGCCATCATGATCT	20	bna-miR167d	1	2	0.0001
	mko-miR167f-3p	ATCATGTGGCAGTTTCACC	21	ptc-miR167f-3p	0	3	0.000002
	mko-miR167h-5p	TGAAGCTGCCAACATGATCTG	21	ptc-miR167h-5p	0	1	0.0000001
	mko-miR167i	TGAAGCTGCCAGCAAGATCTTA	22	mdm-miR167i	1	1	0.000009
MIR168	mko-miR168	TCCCGCCTTGCATCAACTG	24	aau-miR168	0	2	0.000002
	mko-miR168a-3p	CCCGCCTTGCATCAACTGAAT	21	ath-miR168a-3p	0	9	0.0000001
	mko-miR168b	TCGCTTGGTGCAGGTCGGG	19	gma-miR168b	0	9	0.000002
	mko-miR168b-5p	TCGCCTGGTGCAGGTCGGGAA	21	ath-miR168b-5p	1	2	0.00004
MIR169_1	mko-miR169h	TAGCCAAGGATGACTTGCCTG	21	ath-miR169h	0	1	0.0000002
MIR171_1	mko-miR171a	TTGAGCCGCGTCAATATCTCC	21	mdm-miR171a	0	7	0.0000002
MIR172	mko-miR172b	GTGTAGCATCATCAAGAT	21	vvi-miR172b	0	4	0.000009
	mko-miR172b-5p	GCAGCACCATCAAGATTCACA	21	aly-miR172b-5p	0	18	0.0000002
	mko-miR172c-5p	GTAGCATCATCAAGATTCACA	21	mtr-miR172c-5p	0	7	0.0000002
MIR390	mko-miR390a-3p	CGCTATCCATCCTGAGTTTCA	21	ath-miR390a-3p	0	5	0.0000002
	mko-miR390b	AAGCTCAGGAGGGATAGCGCC	21	ppt-miR390b	0	5	0.0000002
MIR393	mko-miR393	CCAAAGGGATCGCATTGATCT	22	ghr-miR393	0	3	0.0000001
MIR396	mko-miR396	TTCCACAGCTTTCTTGAACTT	21	pta-miR396	0	35	0.0000002
	mko-miR396-3p	GCTCAAGAAAGCTGTGGGA	21	ama-miR396-3p	0	2	0.000002
	mko-miR396a	CACAGCTTTCTTGAACTT	21	hbr-miR396a	0	1	0.000009
	mko-miR396a-3p	GTTCAATAAAGCTGTGGGAAG	21	ath-miR396a-3p	0	261	0.0000003
	mko-miR396a-3p	TTCAATAAAGCTGAGGGAAG	20	gma-miR396a-3p	1	1	0.0001
	mko-miR396a-5p	TTCCACAGCTTTCTTGAACTG	21	ath-miR396a-5p	0	1121	0.0000002
	mko-miR396b-3p	GTTCAATAAAGCTGTGGGA	20	osa-miR396b-3p	0	4	0.000003
	mko-miR396c	TTCAAGAAATCTGTGGGAAG	20	csi-miR396c	0	22	0.0000006
	mko-miR396g-3p	CTCAAGAATGCCGTGGGAAA	21	ptc-miR396g-3p	1	3	0.0001
	mko-miR396g-3p	GTTCAAGAAAGCTGTGGAAG	21	zma-miR396g-3p	0	2	0.0000007
	mko-miR396g-5p	TTCCACGGCTTTCTTGAACTT	21	ptc-miR396g-5p	0	144	0.0000001
	mko-miR396j	TTCCACAGCTATCTTGAA	21	gma-miR396j	0	2	0.000009
MIR399	mko-miR399e	CGCCAAAGGAGAGTTGCCCT	21	ptc-miR399e	0	1	0.0000006
MIR403	mko-miR403-3p	TTAGATTCACGCACAAACTCG	21	ath-miR403-3p	0	36	0.0000002
MIR408	mko-miR408-5p	GGGGAACAGGCAGAGCATGG	21	ptc-miR408-5p	0	2	0.0000006
MIR408_2	mko-miR408	ATGCACTGCCTCTTCCCTGGC	21	pta-miR408	0	1	0.0000002
MIR477	mko-miR477a	ACTCTCCCTCAAGGGCTTCTG	21	nta-miR477a	0	6	0.0000002
	mko-miR477a	ATCTCCCTTAAAGGCTTCCAA	21	vvi-miR477a	1	2	0.00003
MIR482	mko-miR472	TTTTCCCACACCTCCCATCCC	21	csi-miR472	0	36	0.0000001
	mko-miR482	TCTTCCCTACTCCACCCAT	22	mes-miR482	0	99	0.000002
	mko-miR482a-3p	TCTTCCCTAAGCCTCCCATTCC	22	csi-miR482a-3p	1	2	0.000009
	mko-miR482b	TCTTGCCCAACCCTCCCATTCC	22	csi-miR482b	1	192	0.000009
	mko-miR482b	TTGCCAACTCCACCCATGCC	22	ghr-miR482b	1	1	0.0001
	mko-miR482c	TTCCCTAGTCCCCCTATTCCTA	22	csi-miR482c	0	138	0.00000004
	mko-miR482d-3p	TCTTCCCTACACCACCCAT	22	gma-miR482d-3p	1	1	0.0005
MIR530	mko-miR530-3p	AGGTGCAGAGGCAGATGCAAC	21	osa-miR530-3p	0	1	0.0000002
MIR535	mko-miR535	TGACAATGAGAGAGAGCAC	21	csi-miR535	0	422	0.000005
	mko-miR535b	TGACAAAGAGAGAGAGCACGC	21	mdm-miR535b	1	3	0.00003
	mko-miR535d	TGACGATGAGAGAGAGCACGC	21	mdm-miR535d	1	1	0.00004
	mko-miR535d	TGACAACGAGAGAGAGCACGC	21	ppt-miR535d	0	1	0.0000001
MIR827	mko-miR827	TTTGCTGATTGTCATCTAA	21	osa-miR827	1	1	0.0006
	mko-miR827-5p	TTTGTTGATTGTCATCTAA	22	bdi-miR827-5p	1	16	0.0006
MIR827_2	mko-miR827b	TTAGATGACCATCAACAAACA	21	ghr-miR827b	0	21	0.0000002
MIR845_2	mko-miR845e	TGGCTCTGATACCAATTGATG	21	vvi-miR845e	0	1	0.0000002
MIR845_3	mko-miR845b	GCTCTAATACCAATTGATA	21	vvi-miR845b	1	3	0.0006
MIR858	mko-miR858	CTCGTTGTCTGTTCGACCTTG	21	ppe-miR858	0	82	0.0000002
MIR1446	mko-miR1446	AACTCTCTCCCTCATAGGCT	21	gra-miR1446	1	5	0.0001
MIR1507	mko-miR1507-3p	CCTCGTTCCAAACATCATCT	22	mtr-miR1507-3p	1	3	0.0001
MIR2673	mko-miR2673b	GAAGAGGAAGAGGAAGAGG	22	mtr-miR2673b	0	1	0.000005
MIR3630	mko-miR3630-3p	TGGGAATCTCTCTGATGCA	22	vvi-miR3630-3p	0	2	0.000004
MIR8654	mko-miR8654c	AGGATACTGCTTTGATGGA	24	gra-miR8654c	1	2	0.0007
MIR9560	mko-miR9560a-5p	CAGGAGGTGGAACAAATATGA	24	bra-miR9560a-5p	1	19	0.00004
NA	mko-miR156i	GACAGAAAAGAGAGAGCAG	20	ath-miR156i	1	1	0.0005
	mko-miR477b	CTCTCCCTCAAGGGCTTCT	21	nta-miR477b	0	2	0.000002
	mko-miR477h	ACTCTCCCTCAAGGGCTTCA	21	mes-miR477h	0	1	0.0000006
	mko-miR845	GCTCTGATACCAATTGTTG	21	bdi-miR845	0	4	0.000003
	mko-miR894	CGTTTCACGTCGGGTTCACC	20	ppt-miR894	0	2	0.0000006
	mko-miR1446	TTCTAAACTCTCTCCCTCAT	20	mes-miR1446	1	3	0.0001
	mko-miR1515	ATTTTTGCGTGCAATGATCC	22	csi-miR1515	0	2	0.0000006
	mko-miR2916	TGGGGGCTCGAAGACGATCA	23	peu-miR2916	1	13	0.0003
	mko-miR3711	GCCCTCCTTCTAGCGCCA	20	pab-miR3711	0	23	0.00002
	mko-miR3948	TGGAGTGGGAGTGAGAGTA	24	csi-miR3948	1	1	0.0006
	mko-miR3950	AGAAATCATGTTGCAGAAA	21	csi-miR3950	1	1	0.0006
	mko-miR3952	TGAAGGGCCTTTCTAGAGCAC	21	csi-miR3952	0	8	0.0000002
	mko-miR3954	TGGACAGAGAAATCACGGTCA	21	csi-miR3954	0	19	0.0000001
	mko-miR4995	AGGCAGTGGCTTGGTTAAGGG	21	gma-miR4995	0	1	0.0000003
	mko-miR5021	GAGAAGAAGAAGAAGAAAA	20	ath-miR5021	0	1	0.000002
	mko-miR5072	TTCCCCAGTGGAGTCGCCA	22	osa-miR5072	1	2	0.0006
	mko-miR5082	ATGATGGCCTCGCGGGTTCA	24	osa-miR5082	1	47	0.0002
	mko-miR5083	AGACTACAATTATCTGATCA	20	osa-miR5083	0	27	0.000001
	mko-miR5368	GGACAGTCTCAGGTAGACA	19	gma-miR5368	0	592	0.000005
	mko-miR5523	CTAGTAAATACGTTCCTCCTCA	22	osa-miR5523	1	22	0.00002
	mko-miR5532	ATGGAATATATGACAAGGGTGG	22	osa-miR5532	1	2	0.00002
	mko-miR5538	GCAGCAAGTGATTGAGTTCAGT	22	osa-miR5538	0	2	0.00000009
	mko-miR5658	ATGATGAGGATGATGATGAA	21	ath-miR5658	1	2	0.0003
	mko-miR5817	GAATTTGAAAAAAAAAGGT	24	osa-miR5817	1	2	0.0009
	mko-miR6173	AGCCGTAAACGATGGATACT	20	hbr-miR6173	0	3	0.000001
	mko-miR6300	GTCGTTGTAGTATAGTGG	18	gma-miR6300	0	803	0.00002
	mko-miR6478	CCGACCTTAGCTCAGTTGGT	21	ptc-miR6478	0	9	0.000001
	mko-miR6483	TATTGTAGAAATTTTCGGGATC	22	hbr-miR6483	1	19	0.00002
	mko-miR6485	TAGGATGTAGAAGATCATAA	20	hbr-miR6485	1	4	0.0003
	mko-miR7767-5p	CCCCAAGATGAGAGCTCTCC	21	bdi-miR7767-5p	1	1	0.0003
	mko-miR8051-5p	TGAATCTTTATACCATACTA	20	stu-miR8051-5p	1	14	0.0003
	mko-miR8175	GATCCCCGGCAACGGCGCCA	20	ath-miR8175	0	661	0.000001
	mko-miR8610.1	TTTTCTGAACAAATCGAAGAA	24	atr-miR8610.1	1	44	0.00009
	mko-miR9774	GAAATACCCAATATCTTG	22	tae-miR9774	0	1	0.000009

**Table 3:** Potential novel miRNA candidates identified from *M. koenigii*.

Name	Sequence (5′-3′)	Length	Read Count	Strand	MFEI of Precursor
mko-miRN1-3p	UUAGGGUUUCAGUGAUCGAAAAC	23	14	−	0.85
mko-miRN2-3p	GUGAGCCAAGCAAGUAGUGUCGC	23	5	+	0.70
mko-miRN3-3p	UUGUCUCACUGCCUGUUGCACU	22	7	+	0.92
mko-miRN4-5p	UGCAGGUGAGAUGAUACCGUCA	22	15	−	0.72
mko-miRN5-3p	ACCGUGUUUCUCUGCCCAAUCAG	23	8	−	0.98
mko-miRN6-5p	CUGGGGAGUUGCACCCGGAGUA	22	6	−	0.79
mko-miRN7-3p	UUGUUUUGGGUGAAACGGGUGUU	23	93	+	0.97

## Data Availability

Publicly available datasets were analyzed in this study. This data can be found here: https://www.ncbi.nlm.nih.gov/sra/SRR16796893, assessed on 10 June 2021.
